# Balint group work in medical education in China: a quantitative analysis

**DOI:** 10.1186/s12909-026-08726-6

**Published:** 2026-02-05

**Authors:** Jiarui Li, Yanping Duan, Lili Shi, Jinya Cao, Yinan Jiang, Wenqi Geng, Jiaojiao Hu, Chunfeng Xiao, Tao Li, Jing Wei

**Affiliations:** Department of Psychological Medicine, Peking Union Medical College Hospital, Chinese Academy of Medical Sciences and Peking Union Medical College, Shuaifuyuan 1, Dongcheng District, Beijing, 100730 People’s Republic of China

**Keywords:** Balint group, Medical education, Reliability and validity, Quantitative analysis

## Abstract

**Background:**

Balint group work have been increasingly integrated into medical education worldwide. However, quantitative evaluations of their application in medical education using specialized instruments remain limited. This study aimed to quantitatively evaluate the application of Balint group work in medical education in China by assessing students’ perceived learning outcomes and group processes using the Balint Group Questionnaire (BGQ), and to examine factors associated with BGQ scores.

**Methods:**

The Balint group work was embedded within a mandatory medical clinical communication course for postgraduate students. A total of 284 eligible students were approached, and 139 students (48.9%) provided complete questionnaire data and were included in the final analysis. Students could participate in up to six sessions of Balint group work. The BGQ was administered after each session to assess perceived effectiveness, operationalized as BGQ subscale scores reflecting reflective learning and group dynamics. Reliability and validity of the BGQ in this student population were evaluated. Correlation and subgroup analyses were conducted to identify factors associated with BGQ scores.

**Results:**

Cronbach’s alpha coefficients indicated good internal consistency of the BGQ. Confirmatory factor analysis supported a three-factor model with acceptable model fit. Students with fewer years of clinical work experience reported higher BGQ scores across all subscales. A higher frequency of serving as a case reporter was positively associated with BGQ scores, particularly among students with ≥2 years of work experience.

**Conclusions:**

The BGQ demonstrated good reliability and validity for assessing students’ perceived learning outcomes in Balint group work within medical education. Associations between BGQ scores, clinical experience, and case reporter roles suggest that early introduction of Balint groups and active engagement as case reporters may enhance perceived learning benefits.

**Supplementary Information:**

The online version contains supplementary material available at 10.1186/s12909-026-08726-6.

## Introduction

 The Balint group work, developed by Michael Balint, aims to enhance understanding of the doctor-patient relationship and improve treatment dynamics [1, 2]. In these groups, clinicians convene regularly to review cases from their practices, focusing on the psychological aspects of their work, particularly the doctor-patient relationship [3]. Introduced nearly 70 years ago, the Balint group work has been adopted in over 25 countries for clinical work and medical education [4]. In China, the Balint group work has been utilized for almost two decades [5]. Similar to its impact elsewhere, it has effectively mitigated burnout and bolstered self-efficacy among Chinese healthcare professionals [6–8]. During the COVID-19 pandemic, it played a crucial role in supporting the mental health of medical teams as part of broader psychological health initiatives [9]. Globally, the Balint group is widely integrated into medical education, with research indicating its potential to foster patient-centered care, enhance communication skills, and reduce burnout among medical students and residents [10]. In China, there are still few researches on the application of the Balint group work in medical education.

The analysis of Balint group work can be categorized into quantitative and qualitative approaches. Qualitative research methods applied to Balint groups include autoethnography [11], focus group discussions [4], participant observation, leaders’ observations, students’ reflective essays, among others [12]. These qualitative analyses are usually exploratory and require further quantitative analysis. In terms of quantitative analysis, studies examining Balint group work have varied significantly in their outcome measures, resulting in diverse findings [13]. Therefore, the development of a scale specifically tailored to assess the processes within Balint group work becomes crucial. The Balint Group Questionnaire (BGQ) serves as one such tool, validated for reliability and validity in both Germany and China [14]. Nevertheless, evidence regarding its psychometric performance and application in medical student or postgraduate student populations remains scarce.

Accordingly, the objectives of this study were twofold: (1) to evaluate the reliability and validity of the BGQ in a postgraduate medical student population, and (2) to explore social demographic factors associated with BGQ scores as indicators of students’ perceived learning outcomes in Balint group work participation.

## Materials and methods

### Study design and participants

The medical clinical communication program at Peking Union Medical College was a mandatory course for second-year postgraduate students from March 2022 to April 2023. Participation in sessions of Balint group work and completion of questionnaires were voluntary.

Inclusion criteria were: (1) second-year postgraduate students enrolled in the medical clinical communication program, and (2) voluntary participation in the sessions of Balint group work and questionnaire completion. Exclusion criteria were: (1) failure to complete any session of Balint group, and (2) incomplete questionnaire data.

### Balint group procedure

The course spanned a total of 36 class hours, conducted every Saturday over four consecutive weeks. As part of the curriculum, six sessions of Balint group were conducted during the course, with six parallel groups per session. Each group consisted of 7–8 students and was led by an attending physician or senior psychologist with over five years of clinical experience and formal Balint group leadership training. A standardized operational protocol was followed across all groups: (1) A volunteer case reporter briefly presented a clinically challenging case focusing on the doctor-patient relationship, without revealing exhaustive medical details. (2) Group members asked clarifying questions. (3) The group then explored the feelings and behaviors of both the patient and the doctor in the case, with the leader facilitating the discussion using open-ended questions (e.g., “What might the patient be feeling?” “What was the most difficult part of this encounter for you, doctor?”). (4) The case reporter listened without intervening. (5) In the final step, the case reporter shared their reflections on the discussion.

### Data collection and measurement

Sociodemographic data and professional specialization of the participants were collected.

The BGQ was designed as a short questionnaire that could be used in Balint group work research, which records relevant dimensions of Balint group work and is capable of reproducing learning and change processes in future studies with repetitive measurements using operationalized parameters. There were three scales in the BGQ: Scale 1: Reflection of transference dynamics in the doctor-patient relationship (items 2, 10, 13, 15, and 16); Scale 2: Emotional and cognitive learning (items 5, 6, 9, and 11); and Scale 3: Case mirroring in the dynamic of the group (items 4, 7, and 12). The scores reflect the participants’ perceived learning outcomes and group processes. Each item is rated on a six-point Likert scale ranging from 0 (“does not apply”) to 5 (“applies completely”). The questionnaire was firstly designed as a German version, with a good to very good reliabilities (The Cronbach’s alpha of the three scales was between 0.71 and 0.82) and a good to very good model fit (comparative fit index (CFI) = 0.97, root mean square error of approximation (RMSEA) = 0.054, RMR (Root Mean square Residual) = 0.033). The Chinese version of the BGQ also had a good to very good reliability (Cronbach’s alpha = 0.70 to 0.86) and a good model fit for the three-factor model [14]. Formal permission to use the Chinese version of the BGQ for this study was obtained from Professor Kurt Fritzsche. The full items of the BGQ are provided in Supplementary File 1.

Questionnaires were distributed by group leaders via a QR code immediately after the end of each Balint group session. All participants received standardized instructions and completed the questionnaire within a fixed time window (approximately 10–15 min) in the same classroom environment to reduce measurement bias.

### Bias

Possible bias could arise from a trend toward a socially desirable response. This was prevented by the group leaders distributing the questionnaires after the end of the session. The participants were instructed by the group leader to fill out the questionnaires as honestly as possible and the results of the questionnaire will not affect the course grades. To further reduce measurement bias, questionnaire administration procedures, instructions, timing, and completion environment were standardized across all groups and sessions.

### Study size

The sample size calculation followed established guidelines, often referred to as ‘rules of thumb’ or ‘blue-chips’. These guidelines suggest that the ratio of participants (N) to measured variables (p) should ideally range from 5 (with *N* > 100) to 10. A widely accepted benchmark is a minimum of 10 cases per indicator variable [15]. Based on these principles, a total sample size of at least 120 was determined. The Kaiser-Meyer-Olkin (KMO) measure was used to assess sampling adequacy.

### Statistical analysis

The study was structured into two main parts. Firstly, the reliability and validity of the BGQ were evaluated using a sample of students. Secondly, the quantitative analysis focused on factors associated with students’ Balint group work based on BGQ scores.

Continuous variables are presented as mean ± standard deviation. Categorical variables were presented as numbers with percentages. Student’s t-test and one-way analysis of variance (ANOVA) with Scheffé’s post-hoc test (for three or more groups) were used as appropriate to detect any differences in the scores concerning sociodemographic factors.

The data distribution and correlation coefficient with the scale score were calculated for item analysis. Since there were ceiling effects for several items, bootstrap was used in the confirmatory factor analysis (CFA) using AMOS 26.0. An RMR < 0.05, RMSEA value ≤ 0.10, with CFI, normed fit index (NFI), non-normed fit index (NNFI), incremental fit index (IFI), and Tucker–Lewis index (TFI) values > 0.9 were used to identify appropriate global model fits.

To test the internal consistency, Cronbach’s α coefficients were computed for the three scales of BGQ, and 95% confidence intervals (CI) are provided. A Cronbach’s α coefficient above 0.7 indicates high internal consistency, while a value above 0.9 implies redundancy.

The related factors of BGQ were screened by univariate analysis. When the sociodemographic characteristic was the categorical variable, the intergroup differences in BGQ were calculated for different classifications by ANOVA. When the sociodemographic characteristic was a continuous variable, the Pearson’s correlation coefficient between the BGQ and the variable was calculated. The correlation coefficients between the BGQ score and the scores of the other scales were also calculated by Pearson’s correlation coefficient. The ordinal logistic regression analyses were calculated for key relationships where the dependent variable (BGQ score) suffers from ceiling effects. The p values after Bonferroni correction were reported for multiple tests. Analysis of covariance (ANCOVA) was used to analyze covariables and interactions.

## Results

### Characteristics of the participants

**Fig. 1:**
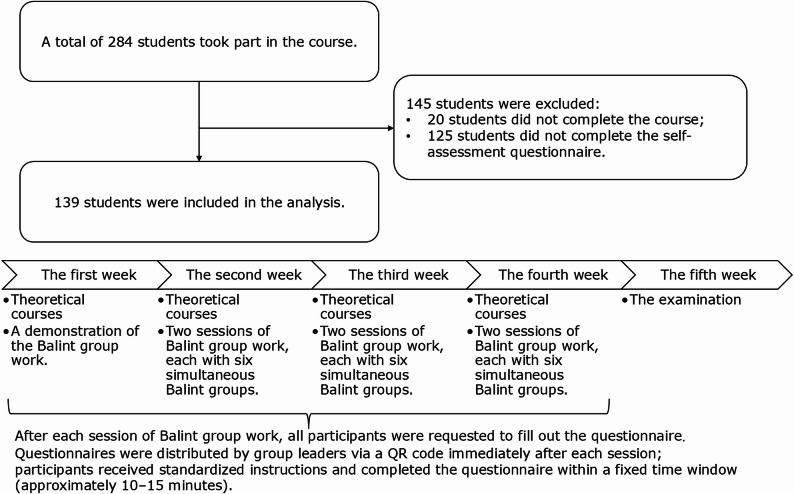
The flow chart

The target population was the entire cohort of second-year postgraduate students (*n* = 284) at our institution during the study period. As the medical clinical communication program was mandatory for this cohort, all 284 students were considered eligible for study inclusion. 139 students included in the final analysis as depicted in Fig. 1. There were no significant differences among the available baseline characteristics (Gender, Department/Surgical vs. Non-surgical, Work Experience) between the included (*n* = 139) and excluded (*n* = 145) participants (Supplementary Table 1). Their mean age was 25.15 ± 1.50 years, and 48 (34.53%) were male. Participants were distributed across non-surgical wards (*n* = 80, 38.46%), surgical wards (*n* = 84, 40.38%), and other departments (*n* = 44, 21.15%), with a median professional experience of 2 (range 0–6) years. A significant majority, 130 (93.53%) students, participated in Balint group sessions 3–6 times. Additionally, 88 (63.31%) students did not serve as case reporters in any Balint group sessions.

The scores for the three BGQ subscales were as follows: Scale 1: 4.00 ± 0.74, Scale 2: 3.85 ± 0.78, and Scale 3: 3.92 ± 0.73. There were no significant differences observed among the scores of these subscales (*p* > 0.05). The high BGQ scores across subscales indicate favorable participant evaluations of perceived learning and group processes. As this was a cross-sectional post-test design, these scores reflect perceptions at a single time point and not longitudinal change.

### The reliability and validity of the BGQ

Supplementary Table 2 presents the characteristics of the items. All items exhibited a significant ceiling effect, with a median score of 4. The pronounced ceiling effects observed suggest that the BGQ, in its current form, may have limited ability to discriminate among participants who already possess high levels of reflective ability. This compression of scores at the upper end of the scale could potentially attenuate the strength of the correlations observed in subsequent analyses.

Item distributions were unimodal and non-normally distributed, with skewness ranging from − 1.16 to -0.5 and kurtosis ranging from − 0.53 to 1.88. Across the three scales, the correlation coefficients between item scores and scale scores were as follows: Scale 1 (r: 0.823–0.908), Scale 2 (r: 0.872–0.909), and Scale 3 (r: 0.780–0.878) (*p* < 0.01).

**Fig. 2:**
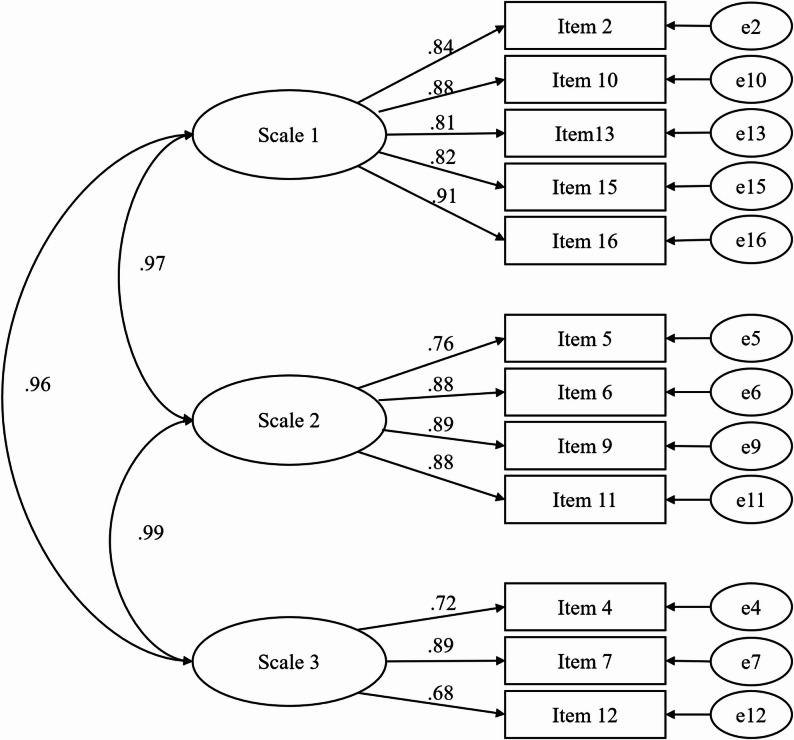
The model of structural validity

Due to the non-normal distribution of the data, confirmatory factor analysis of the three-factor model was conducted using the bootstrap method with 200 iterations in AMOS. Figure 2 displays the load factors for each item. The model demonstrated good fit indices: RMR 0.038, RMSEA 0.087, CFI 0.947, NFI delta1 0.918, IFI delta2 0.948, and TLI rho2 0.932, indicating good to very good model fit.

A reliability analysis based on the three-factor model indicated satisfactory internal consistency of the instrument. Cronbach’s alpha coefficients were as follows: Scale 1 α = 0.924, Scale 2 α = 0.911, and Scale 3 α = 0.795, indicating good internal consistency reliability for each scale.

### The related factors of the BGQ

The scores of the three BGQ scales and the total BGQ score did not show significant differences between sexes (*p* > 0.05) or among surgical, non-surgical, and other departments (*p* > 0.05). Similarly, BGQ scores were not significantly associated with age or the frequency of participation in Balint group sessions (*p* > 0.05).

There was a significant negative correlation between years of work experience and scores on all three BGQ subscales (Scale 1: *r* = -0.175, *p* = 0.040 < 0.05; Scale 2: *r* = -0.184, *p* = 0.030 < 0.05; Scale 3: *r* = -0.188, *p* = 0.026 < 0.05). Additionally, a significant positive correlation was found between the frequency of being a case reporter and average BGQ scores (Scale 1: *r* = 0.172, *p* = 0.010 < 0.05; Scale 2: *r* = 0.198, *p* = 0.003 < 0.01; Scale 3: *r* = 0.220, *p* = 0.001 < 0.01). Among the 41 students who reported cases, scores on Scale 1 (4.32 ± 0.62 vs. 4.09 ± 0.79, *p* = 0.017 < 0.05) and Scale 2 (4.23 ± 0.72 vs. 4.03 ± 0.84, *p* = 0.029 < 0.05) were significantly higher when they acted as case reporters compared to when they did not, although the difference in Scale 3 was not significant (4.24 ± 0.71 vs. 4.06 ± 0.80, *p* = 0.057 > 0.05). To account for the ceiling effects in BGQ scores, ordinal logistic regression was additionally performed. This analysis confirmed a significant positive association between the frequency of being a case reporter and higher scores on Scale 1 (OR = 1.82, 95% CI [1.15, 2.89], *p* = 0.011 < 0.05), Scale 2 (OR = 2.12, 95% CI [1.32, 3.41], *p* = 0.002 < 0.01), and Scale 3 (OR = 1.90, 95% CI [1.19, 3.02], *p* = 0.007 < 0.01).

Students were categorized into two groups based on work experience: Subgroup A (less than 2 years, *n* = 53) and Subgroup B (2 or more years, *n* = 86). There were no significant differences between the two groups in terms of frequency of Balint group participation or being as a case reporter (*p* > 0.05). In Subgroup B, there was a significant positive correlation between the number of case reports and scores on all three BGQ subscales, whereas no such correlation was found in Subgroup A (see Table 1 for details).

**Table 1:** The correlation coefficient between the balint group questionnaire score and other relevant factors

	The Scale 1 of the BGQ	The Scale 2 of the BGQ	The Scale 3 of the BGQ
Total (*n* = 139)			
Years of work experience	-0.175*	-0.184*	-0.188*
Frequency of participation in Balint group	0.140	0.117	0.127
Frequency of being case reporter	0.199*	0.245**	0.200
Subgroup A (*n* = 53)			
Years of work experience	0.036	-0.008	0.058
Frequency of participation in Balint group	0.073	0.003	0.018
Frequency of being case reporter	0.009	-0.008	-0.064
Subgroup B (*n* = 86)			
Years of work experience	-0.283**	-0.244**	-0.316**
Frequency of participation in Balint group	0.176	0.172	0.197
Frequency of being case reporter	0.297**	0.370**	0.341**
*p<0.05, **p<0.01			

BGQ: Balint Group Questionnaire; Subgroup A: Students with less than 2 years of work experience; Subgroup B: Students with at least 2 years of work experience

## Discussion

A total of 139 students who participated in the Balint Group work course were included in this study, and their progress from the course was assessed using the Balint Group Questionnaire (BGQ). Analysis of the three scale scores demonstrated significant high scores across all measured areas. The BGQ exhibited robust reliability and validity among the students. Confirmatory factor analysis confirmed the structural validity of the three-factor model within the student population. Furthermore, the frequency of being a case reporter and professional work experience were significantly related with BGQ scores. Subgroup analysis revealed a notable correlation between the number of case reports and BGQ scores specifically among students with two or more years of professional experience.

The BGQ is specifically designed to evaluate the Balint group, capturing participants’ immediate perceptions and impressions following sessions to elucidate group dynamics and interpersonal factors. In this study, the structural validity of the three-factor model was reaffirmed among students, referencing the original German version of the BGQ [16]. Scores from the three subscales served as dependent variables in subsequent analyses. Scale 1 assesses “reflection of transference dynamics in the doctor-patient relationship,” encompassing “diagnostics of the doctor-patient relationship” (transference dynamics) and “awareness of one’s own contributions to the doctor-patient relationship.” Scale 2, focused on “emotional and cognitive learning,” pertains to “medical participants’ learning experiences in the doctor-patient relationship.” Scale 3 examines “case mirroring in group dynamics,” reflecting “the group’s reflection on presented patient cases.“^14^ Given the cross-sectional, post-test-only design, BGQ scores should be interpreted as indicators of students’ perceived learning outcomes rather than objective improvements or longitudinal change. Similar to these findings, a five-week study involving medical students in Balint group work found that participants appreciated the opportunity to consider the role of emotions in patient interactions and viewed the groups as beneficial for exploring doctor-patient relationships [17], aligning with the implications of Scale 1 in this study.

The integration of Balint group work into undergraduate curricula in countries like the US, UK, and Australia underscores its global relevance in fostering reflective practice [18–20]. There remains ongoing debate regarding the optimal timing for introducing Balint groups in medical education. Some researchers argue that early introduction during medical training might diminish benefits due to limited clinical experience and patient understanding [20]. Findings from this study indicate a significant negative correlation between years of working experience and BGQ scores, suggesting that students with less clinical experience may derive greater perceived benefits from the Balint group in terms of relational reflection and learning. This supports the recommendation for introducing Balint group work earlier in medical education, for instance, during the initial stages of clinical practice or the first year of postgraduate training, to maximize their potential impact.

Previous research has suggested that younger doctors may require at least 1.5 years of Balint group work participation to derive substantial benefits [4], but more research indicates that medical students feel they have gained something from 4 to 6 sessions of Balint group work [19, 21, 22]. although this study did not find a significant correlation between the frequency of Balint group meetings and BGQ scores. Possible reasons for this disparity include the sensitivity limitations of the BGQ or the relatively short follow-up period in this study. It is also important to note that the Balint groups were embedded within a broader 36-hour clinical communication course. Therefore, the outcomes measured likely reflect the combined effect of the entire curriculum, and the specific contribution of the 6-hour Balint group component cannot be isolated.

While some studies have focused on the impact of group leaders [23], less attention has been given to the experiences of case presenters. The positive association between serving as a case reporter and BGQ scores among more experienced students highlights the potential value of encouraging active participation. Recommendations for early implementation and structured encouragement of case presentation are therefore based on observed associations rather than causal inference. Based on our findings, we recommend the early integration of Balint group work into medical education, ideally at the commencement of clinical rotations or postgraduate training, to capitalize on the greater perceived benefits observed in less experienced students. To support implementation in resource-limited settings, alternatives such as virtual Balint group work, training a core faculty of facilitators through train-the-trainer programs, and utilizing standardized video demonstrations ofsessions of Balint group work should be explored. Future efforts could also explore the development of preparatory materials to support all students in effectively engaging with the Balint group work process.

This study also has some limitations. Firstly, it is a single-center study, limiting its generalizability, and future research should consider multicenter studies. Secondly, this study is a single-post-test design without baseline data. Causal effects of Balint group participation cannot be inferred. Thirdly, the effective response rate was 48.9% (139/284), which may introduce non-response bias and limit the generalizability of our findings. Although we found no significant differences in available baseline characteristics (e.g., gender, department distribution) between included and excluded participants (see Supplementary Table 1), the possibility of bias due to unmeasured factors (e.g., baseline empathy levels) cannot be ruled out. Future studies should implement more robust strategies to maximize participant retention. Finally, as a relatively novel tool, the BGQ requires broader adoption and comparative studies across different contexts to establish its validity and reliability.

## Conclusions

The BGQ is a reliable and valid instrument for assessing perceived learning outcomes and group processes in Balint group work among postgraduate medical students. Early integration of Balint group work and active engagement as case reporters may enhance perceived educational value, although causal relationships require confirmation in future longitudinal studies.

## Supplementary Information

Supplementary Material 1.

## Data Availability

The datasets used and/or analyzed during the current study are available from the corresponding author on reasonable request.

## Abbreviations

ANCOVA: Analysis of covariance
ANOVA: Analysis of variance
BGQ: Balint Group Questionnaire
CFA: Confirmatory factor analysis
CFI: Comparative fit index
CI: Confidence intervals
IFI: Incremental fit index
KMO: Kaiser-Meyer-Olkin
