# Supplementary material for: Balint group work in medical education in China: a quantitative analysis
Source: BMC Med Educ. 2026 Feb 5;26:480. doi: 10.1186/s12909-026-08726-6 (PMC13019891; doi:10.1186/s12909-026-08726-6)
Supplement: Supplementary file 1 — Supplementary Material 1. [file 12909_2026_8726_MOESM1_ESM.docx]

**巴林特小组问卷**

**在今天的巴林特小组活动中 ...** （每个问题只选一个选项）

|  | **内容** | **不符合** | **极少符合** | **有些符合** | **比较符合** | **符合** | **完全符合** |
| --- | --- | --- | --- | --- | --- | --- | --- |
| 1 | 对于患者对我的感受的影响，我思考了很多 | 0 | 1 | 2 | 3 | 4 | 5 |
| 2 | 让我清楚意识到，潜意识的情绪可能对医患关系产生多么强烈的影响 | 0 | 1 | 2 | 3 | 4 | 5 |
| 3 | 小组通过询问案例汇报者在案例中的角色，对他的反应进行了深入细致的工作 | 0 | 1 | 2 | 3 | 4 | 5 |
| 4 | 小组讨论了案例的动力在多大程度上反复影响到小组的氛围 | 0 | 1 | 2 | 3 | 4 | 5 |
| 5 | 通过对案例的工作，我的情绪得到了释放 | 0 | 1 | 2 | 3 | 4 | 5 |
| 6 | 通过小组对案例的工作，我学到了前所未有的新知识 | 0 | 1 | 2 | 3 | 4 | 5 |
| 7 | 我意识到, 案例的动力是多么充分地渗透到小组的工作氛围中 | 0 | 1 | 2 | 3 | 4 | 5 |
| 8 | 组长引导小组在某些特定方面进行思考 | 0 | 1 | 2 | 3 | 4 | 5 |
| 9 | 我获得了看待案例的不同视角，因而对医患关系有了全新认识 | 0 | 1 | 2 | 3 | 4 | 5 |
| 10 | 我认识到，诊疗过程会在多大程度上受到自己无意识的反应的影响 | 0 | 1 | 2 | 3 | 4 | 5 |
| 11 | 对今后与患者的工作，我获得了重要的推动力 | 0 | 1 | 2 | 3 | 4 | 5 |
| 12 | 小组注意到，案例激起了个别一些成员的反应 | 0 | 1 | 2 | 3 | 4 | 5 |
| 13 | 讨论涉及了医生的行为在诊疗过程或医患关系中所起的作用 | 0 | 1 | 2 | 3 | 4 | 5 |
| 14 | 对之前未察觉到的患者的期待及这种期待对我个人诊疗行为的影响，我突然“恍然大悟” | 0 | 1 | 2 | 3 | 4 | 5 |
| 15 | 我意识到，之前未察觉到的情感在多大程度上参与了治疗关系的构建 | 0 | 1 | 2 | 3 | 4 | 5 |
| 16 | 我明白了，自己对患者的看法会对诊疗过程产生多大的影响 | 0 | 1 | 2 | 3 | 4 | 5 |
| 17 | 处理医患关系的工作带给我很多的乐趣 | 0 | 1 | 2 | 3 | 4 | 5 |

为使语言简洁，以上问题中使用“医生”代指所有人（包括医生、心理学家、社工、护士、医院管理人员等）

**Balint Group Questionnaire**

**In today's Balint group session... (Choose only ONE option per question)**

|  |  | **Disagree** |  |  |  |  | **Completely agree** |
| --- | --- | --- | --- | --- | --- | --- | --- |
| 1 | I thought a lot about how the patient's feelings affect me | 0 | 1 | 2 | 3 | 4 | 5 |
| 2 | It made me clearly aware how strongly unconscious emotions can affect the doctor-patient relationship | 0 | 1 | 2 | 3 | 4 | 5 |
| 3 | The group worked intensively and in detail on the presenter's reactions by asking about their role in the case | 0 | 1 | 2 | 3 | 4 | 5 |
| 4 | The group discussed how the case dynamics recurrently affected the group's atmosphere | 0 | 1 | 2 | 3 | 4 | 5 |
| 5 | Working on the case provided me with emotional relief | 0 | 1 | 2 | 3 | 4 | 5 |
| 6 | Through the group's work on the case, I learned something completely new | 0 | 1 | 2 | 3 | 4 | 5 |
| 7 | I realized how fully the case dynamics permeated the working atmosphere of the group | 0 | 1 | 2 | 3 | 4 | 5 |
| 8 | The facilitator guided the group to reflect on specific aspects | 0 | 1 | 2 | 3 | 4 | 5 |
| 9 | I gained different perspectives on the case, leading to a new understanding of the doctor-patient relationship | 0 | 1 | 2 | 3 | 4 | 5 |
| 10 | I recognized the extent to which the diagnostic/therapeutic process can be influenced by one's own unconscious reactions | 0 | 1 | 2 | 3 | 4 | 5 |
| 11 | I gained important impetus for my future work with patients | 0 | 1 | 2 | 3 | 4 | 5 |
| 12 | The group noticed that the case evoked reactions in individual members | 0 | 1 | 2 | 3 | 4 | 5 |
| 13 | The discussion addressed the role of the doctor's behavior in the diagnostic/therapeutic process or the doctor-patient relationship | 0 | 1 | 2 | 3 | 4 | 5 |
| 14 | I suddenly "saw the light" regarding previously unrecognized patient expectations and their influence on my professional actions | 0 | 1 | 2 | 3 | 4 | 5 |
| 15 | I became aware of the extent to which previously unrecognized emotions participate in shaping the therapeutic relationship | 0 | 1 | 2 | 3 | 4 | 5 |
| 16 | I understood how much my perception of the patient can influence the diagnostic/therapeutic process | 0 | 1 | 2 | 3 | 4 | 5 |
| 17 | Working on the doctor-patient relationship brought me a lot of enjoyment | 0 | 1 | 2 | 3 | 4 | 5 |

For the sake of linguistic simplicity, the term "doctor" is used in the above questions to refer to all participants (including physicians, psychologists, social workers, nurses, hospital administrators, etc.).
